# Complex Machine-Learning Algorithms and Multivariable Logistic Regression on Par in the Prediction of Insufficient Clinical Response to Methotrexate in Rheumatoid Arthritis

**DOI:** 10.3390/jpm11010044

**Published:** 2021-01-14

**Authors:** Helen R. Gosselt, Maxime M. A. Verhoeven, Maja Bulatović-Ćalasan, Paco M. Welsing, Maurits C. F. J. de Rotte, Johanna M. W. Hazes, Floris P. J. G. Lafeber, Mark Hoogendoorn, Robert de Jonge

**Affiliations:** 1 Department of Clinical Chemistry, Amsterdam Gastroenterology and Metabolism, Amsterdam UMC, VUmc, 1081 HV Amsterdam, The Netherlands; r.dejonge1@amsterdamumc.nl; 2 Department of Clinical Chemistry, Erasmus MC, University Medical Center Rotterdam, 3015 GD Rotterdam, The Netherlands; 3 Department of Rheumatology & Clinical Immunology, UMC Utrecht, 3508 GA Utrecht, The Netherlands; m.m.a.verhoeven-15@umcutrecht.nl (M.M.A.V.); m.bulatovic@umcutrecht.nl (M.B.-Ć.); p.m.j.welsing@umcutrecht.nl (P.M.W.); f.lafeber@umcutrecht.nl (F.P.J.G.L.); 4 Department of Internal Medicine, UMC Utrecht, 3508 GA Utrecht, The Netherlands; 5 Department of Clinical Chemistry, Amsterdam Gastroenterology and Metabolism, Amsterdam UMC, Univ of Amsterdam, 1105 AZ Amsterdam, The Netherlands; m.derotte@amsterdamumc.nl; 6 Department of Rheumatology, Erasmus MC, University Medical Center Rotterdam, 3015 GD Rotterdam, The Netherlands; j.hazes@erasmusmc.nl; 7 Department of Computer Science, Quantitative Data Analytics Group, Vrije Universiteit Amsterdam, 1081 HV Amsterdam, The Netherlands; m.hoogendoorn@vu.nl

**Keywords:** arthritis, rheumatoid, methotrexate, outcome assessment, healthcare, therapeutics

## Abstract

The goals of this study were to examine whether machine-learning algorithms outperform multivariable logistic regression in the prediction of insufficient response to methotrexate (MTX); secondly, to examine which features are essential for correct prediction; and finally, to investigate whether the best performing model specifically identifies insufficient responders to MTX (combination) therapy. The prediction of insufficient response (3-month Disease Activity Score 28-Erythrocyte-sedimentation rate (DAS28-ESR) > 3.2) was assessed using logistic regression, least absolute shrinkage and selection operator (LASSO), random forest, and extreme gradient boosting (XGBoost). The baseline features of 355 rheumatoid arthritis (RA) patients from the “treatment in the Rotterdam Early Arthritis CoHort” (tREACH) and the U-Act-Early trial were combined for analyses. The model performances were compared using area under the curve (AUC) of receiver operating characteristic (ROC) curves, 95% confidence intervals (95% CI), and sensitivity and specificity. Finally, the best performing model following feature selection was tested on 101 RA patients starting tocilizumab (TCZ)-monotherapy. Logistic regression (AUC = 0.77 95% CI: 0.68–0.86) performed as well as LASSO (AUC = 0.76, 95% CI: 0.67–0.85), random forest (AUC = 0.71, 95% CI: 0.61 = 0.81), and XGBoost (AUC = 0.70, 95% CI: 0.61–0.81), yet logistic regression reached the highest sensitivity (81%). The most important features were baseline DAS28 (components). For all algorithms, models with six features performed similarly to those with 16. When applied to the TCZ-monotherapy group, logistic regression’s sensitivity significantly dropped from 83% to 69% (*p* = 0.03). In the current dataset, logistic regression performed equally well compared to machine-learning algorithms in the prediction of insufficient response to MTX. Models could be reduced to six features, which are more conducive for clinical implementation. Interestingly, the prediction model was specific to MTX (combination) therapy response.

## 1. Introduction

Methotrexate (MTX) is the anchor drug in the treatment of rheumatoid arthritis (RA) patients. Currently, every early RA patient receives MTX (combination) therapy for at least 3–6 months, which is the interval of the build-up dose and to reliably determine the response to MTX [1]. A substantial proportion of patients do not benefit from MTX treatment due to the inefficacy or adverse events and require a step-up treatment with targeted synthetic or biologic disease-modifying antirheumatic drugs (ts/bDMARDs) [1]. Preferably, personalized medicine is implemented, allowing the predicted insufficient responders to MTX a step-up treatment from the start. To enable personalized medicine, we and others have previously proposed prediction models to identify insufficient responders to MTX prior to treatment initiation [2,3,4,5,6]. We recently externally validated our model and implemented it in the online clinical tool *Evidencio* [7]. Until now, these clinical prediction models have been developed using multivariable logistic regression. In recent years, the use of machine-learning algorithms has gained popularity in healthcare due to their flexibility in handling large complex datasets and nonlinear relationships [8,9]. In addition, in the RA healthcare domain there are many opportunities for the application of machine-learning algorithms, for instance, the categorization of different arthritis subtypes or prediction of treatment response [10,11,12]. Others already successfully examined whether machine-learning algorithms could be used to predict response to MTX therapy in juvenile idiopathic arthritis (JIA) patients [13] and to antitumor necrosis factor in RA patients [14]. However, it is unclear whether these algorithms outperform multivariable logistic regression models in the prediction of insufficient response, as these statistical techniques have not been examined in parallel.

To facilitate clinical implementation, an insightful model using the least number of variables, referred to as “features”, is preferred. Several feature selection methods exist to determine the essential features, and some automated feature selection methods are embedded within machine-learning algorithms (e.g., least absolute shrinkage and selection operator (LASSO)) [15]. Furthermore, it is essential to predict insufficient response specifically to MTX (combination) therapy because these patients would benefit from a step-up treatment, while other strategies are required for RA patients that are also irresponsive to nonconventional DMARDs (e.g., toculizumab (TCZ)) [16].

On the basis of the points described above, the primary aim of this study was to assess the performance of machine-learning algorithms compared to multivariable logistic regression in prediction of insufficient response to MTX (combination) therapy in RA patients. Secondly, feature selection was performed to examine which features are essential to predict insufficient response in RA. Lastly, to investigate whether a model identifies insufficient responders specifically to MTX (combination) therapy, the best-performing model was also assessed on a similar group of RA patients starting TCZ-monotherapy.

## 2. Materials and Methods

### 2.1. Patients

Three hundred and fifty-five subjects were included in current study. Two hundred and sixty-four patients were randomized to start MTX monotherapy or MTX combination therapy with conventional DMARDs (i.e., sulfasalazine (SSZ) and hydroxychloroquine (HCQ)) and corticosteroids, satisfying the 2010 American college of Rheumatology (ACR)/European League Against Rheumatism (EULAR) classification criteria for RA. Those whose Disease Activity Score 28 (DAS28) was available at 3 months were eligible for the treatment in the Rotterdam Early Arthritis Cohort (tREACH, registered retrospectively at ISRCTN, registry number: ISRCTN26791028 on 23 August 2007), as well as 91 patients from the U-Act-Early trial registered at ClinicalTrials.gov (number: NCT01034137).

The tREACH described previously [17] was designed to achieve early response rates (within 3 months), by quickly increasing MTX dosage up to 25 mg/week within the first 3 weeks. U-Act-Early, also previously described [18], consisted of three treatment arms: MTX + placebo, TCZ + MTX, and TCZ + placebo. MTX dosage was increased 5 mg per 4 weeks up to 30 mg/week with a starting dose of 10 mg/week, and the use of corticosteroids was not permitted. Ninety-one patients of the MTX-monotherapy arm and 101 RA patients from the TCZ-monotherapy arm of U-Act-Early were included in the current study. Two patients from the total TCZ arm (*N* = 103) were excluded from the analyses due to missing DAS28 scores at 3 months.

U-Act-Early was approved by the medical ethics committee of the University Medical Center Utrecht (ML22497) and the tREACH by the medical ethics committee of Erasmus Medical Center Rotterdam (MEC-2006-252). Written informed consent was obtained for all included patients.

### 2.2. Features and Outcome

Features related to RA pathogenesis (rheumatoid factor (RF), anticitrullinated protein antibody (ACPA) status, and DAS28 components) or to MTX metabolism (e.g., single nucleotide polymorphisms (SNPs) in ATP-binding cassette (ABC) transporter genes and erythrocyte folate) that were available in both the tREACH and U-Act-Early are presented in Table 1. The outcome “insufficient response” was defined as DAS28 > 3.2, based on the erythrocyte sedimentation rate (ESR), and was determined at 3 months, because after that point in time, treatment could be intensified with a bDMARD in the tREACH.

### 2.3. Training and Test Data

The total dataset contained 355 subjects and 16 features (Table 1). The data were first split into a training (70%, *N* = 249) and a test set (30%, *N* = 106). A stratified split was applied, meaning that the ratio between insufficient and sufficient responders was kept similar to the ratio in the complete dataset. Hence, the training set contained *N* = 124/249 insufficient responders (50%), and the test set contained *N* = 53/106 insufficient responders (50%) at 3 months. Moreover, the training and test sets were fixed upfront using a random seed. To prevent data leakage, preprocessing steps were performed on the training and test sets separately. At the start, all features contained <20% missing values. Missing values were imputed using K-nearest neighbors, a widely used technique where imputation is based on the values of other patients (neighbors) with the most similar data [19]. To prevent ties in imputation of categorical features, only odd numbers (K = 3, 5, 7, 9, 11) were tested. K = 5 was initially randomly chosen and showed comparable results to the other K values and was therefore used for imputation. All analyses were performed in RStudio Version 1.3.1056.

### 2.4. Algorithms, Preprocessing, and Statistics

Mean baseline characteristics between insufficient and sufficient responders to MTX (combination) therapy in the complete dataset were compared using a Welch’s two-sample t-test, and proportions were compared using the two-sample test for equality of proportions. The following random selection of popular supervised classification algorithms were tested and compared to logistic regression for the prediction of insufficient response: least absolute shrinkage and selection operator (LASSO) [20], random forest, and extreme gradient boosting (XGBoost) [21,22]. The latter two algorithms are based on decision trees. Preprocessing for LASSO included centering and scaling of the features. We performed 10-fold stratified cross-validation to tune the hyperparameters to avoid overfitting. Hyperparameters were automatically tuned [23] and the best hyperparameters of the final models were random forest (mtry = 4, ntree = 500), LASSO (alpha = 1, 0.017), and XGBoost (eta = 0.3, max_depth = 1, gamma = 0, colsample_bytree = 0.6, min_child_weight = 1, subsample = 0.67).

First, the model performances on the training set (70%) were assessed using the area under the curve (AUC) of the receiver-operating characteristic (ROC) curves. Second, the performances of the tuned models were examined on the test set. A random seed was again set to make the model assessments reproducible. The differences between two ROC curves were tested using DeLong’s test. Additionally, accuracy, precision, sensitivity, specificity, negative predictive value (NPV), and positive predictive value (PPV) were assessed using the pROC package [24]. A cut-off was chosen based on the highest possible sensitivity and specificity of ≥0.60. The rationale behind this step was the correct identification of as many insufficient responders as possible (sensitivity), while maintaining the correct classification of sufficient responders (specificity). The differences in sensitivity were tested using a 2-sample test for equality of proportions with continuity correction. Third, feature selection was performed in order to simplify the models for clinical application. To determine the essential features for prediction of insufficient response, feature importance plots were created based on their regression coefficients (logistic/LASSO) or decrease in accuracy/Gini score (random forest/XGBoost). Additionally, feature correlations were examined using Pearson’s correlation test. In case of two highly correlated features (r > 0.60), the feature that was easiest to clinically assess was included. Finally, the best-performing model was applied to a TCZ-monotherapy group, and its performance was compared to the performance on the MTX (combination) therapy group (for which it was developed). First, power calculations for the AUCs were performed using the pROC package in R to assure that enough cases were included [25]. Next, calibration curves were generated for the two treatment groups (i.e., MTX combination therapy or TCZ-monotherapy) in order to examine the concordance between the calculated (using model) and observed probabilities of insufficient response. Furthermore, to compare the model’s fit on the MTX (combination) therapy group and TCZ-monotherapy group, a risk score for insufficient response was calculated according to the logistic model coefficients (intercept + β_1_ × pred1 + β_2_ × pred2, etc.). To compare the differences between the two calibration curves, the main effects “risk score” and “treatment group” and their interaction term were assessed in relation to the prediction of insufficient response in a logistic regression model on the total dataset (MTX combination therapy + TCZ, *N* = 435), excluding cases with incomplete values for any feature.

## 3. Results

### 3.1. Baseline Comparisons

Our data were balanced with 49.9% insufficient responders (DAS28 > 3.2) after 3 months of treatment and 50.1% sufficient responders (Table 2). The majority received combination therapy with SSZ, HCQ, and/or corticosteroids. Significantly more patients on MTX-monotherapy (*p* = 0.01) and on MTX combination therapy with intramuscular corticosteroids (*p* = 0.04) were insufficient responders.

### 3.2. Model Performances on Test Set—Including All Features

Performances between tuned algorithms on the training set were comparable with AUCs ranging from 0.71 to 0.73 (Supplemental Table S1). Next, trained models were tested on the test set (*N* = 106). The highest AUC of 0.77 (95% CI: 0.68–0.86) was reached with logistic regression (Table 3).

**Figure 1:**
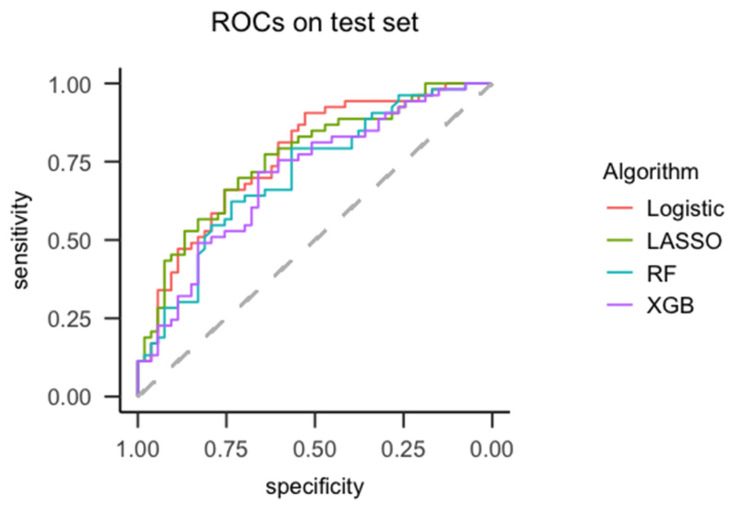
Receiver-operating characteristic (ROC) curves of algorithms tested on test set (*N* = 106). Abbreviations: RF = Random forest, Logistic = logistic regression, XGB = Extreme gradient boosting, LASSO = least absolute shrinkage and selection operator.

Largest differences in AUCs were observed between logistic regression and random forest (Figure 1), although these were not significantly different (*p* = 0.09).

Sensitivity was significantly higher in logistic regression (*p* = 0.02) and borderline significantly higher in LASSO (*p* = 0.05) compared to random forest (Table 3). A sensitivity of 0.81 (logistic regression) indicates that 81% of all insufficient responders were correctly identified as such. The PPV, indicating percentage of predicted insufficient responders that were true insufficient responders, was comparable between algorithms.

### 3.3. Feature Importance

**Figure 2:**
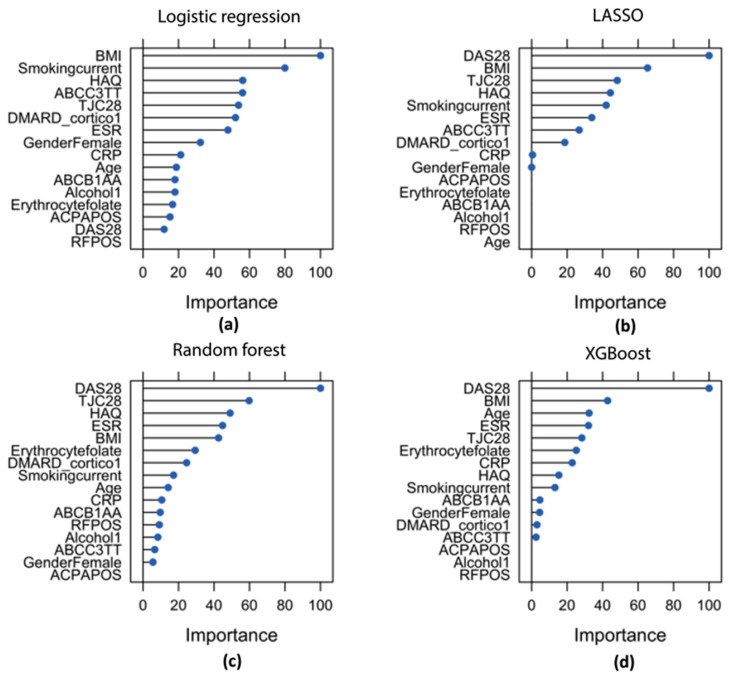
Feature importance plots of baseline features for (**a**) logistic regression, (**b**) LASSO, (**c**) random forest, and (**d**) XGBoost in the prediction of insufficient response at 3 months. Feature importance was determined based on regression coefficients (regression models) and the Gini score (RF and XGBoost) of final models. The most important feature was set to 100, and the rest is relative to that feature. Abbreviations: DAS28 = disease activity score 28, TJC28 = tender joint count 28, HAQ = Health Assessment Questionnaire, ESR = erythrocyte sedimentation rate, BMI = body mass index, DMARD_cortico1 = use of DMARDs or corticosteroids (0 = no, 1 = yes), Smoking (never/former versus current), CRP = c-reactive protein, ABCB1 AA vs. AG/GG, ABCC3 TT vs. TC/CC, RF = rheumatoid factor, Alcohol use (0 = 1 no, 1 = yes), ACPA = anticitrullinated protein (positive versus negative).

Features’ contributions to the model performances are presented in Figure 2. Features that were important for all algorithms were baseline DAS28 or DAS28 components (Tender Joint Count 28 (TJC28), ESR/C-reactive Protein (CRP), Health Assessment Questionnaire (HAQ)). Depending on the algorithm, current smoking, erythrocyte folate, ABCC3 genotype, BMI, and the use of DMARDs/corticosteroids were important features in the identification of insufficient responders. RF positivity, ACPA positivity, and alcohol use were the least important for the majority of the algorithms. Of all the algorithms, LASSO performed the most rigorous feature selection, selecting DAS28, HAQ, TJC28, smoking, ESR, ABCC3 genotype, DMARD/corticosteroid use, CRP, and gender. However, gender and CRP were less important compared to the other selected features.

### 3.4. Feature Selection

Feature selection was performed to boost the model performances and retrieve more clinically applicable concise models. We started from the features selected by LASSO. Additionally, we excluded one out of two highly correlated features, e.g., DAS28 and TJC28 (r = 0.73) and CRP and ESR (r = 0.61). TJC28 requires fewer clinical assessments compared to DAS28, and the outcome was based on DAS28-ESR, which is why TJC28 and ESR were chosen. Even though the ABCC3 genotype was selected by LASSO, we excluded this feature because of its absence in the TCZ-monotherapy group and its minor contribution compared to the other features. Hence, features included after selection were TJC28, HAQ, BMI, smoking, ESR, and the use of DMARDs/corticosteroid use. All models performed equally well with only six features (Table 4) compared to the complete set of features (=16 features; Table 1). The ROCs are presented in Supplemental Figure S1 and confusion matrices in Figure S2.

### 3.5. Model Assessment on TCZ-Monotherapy Arm

**Figure 3:**
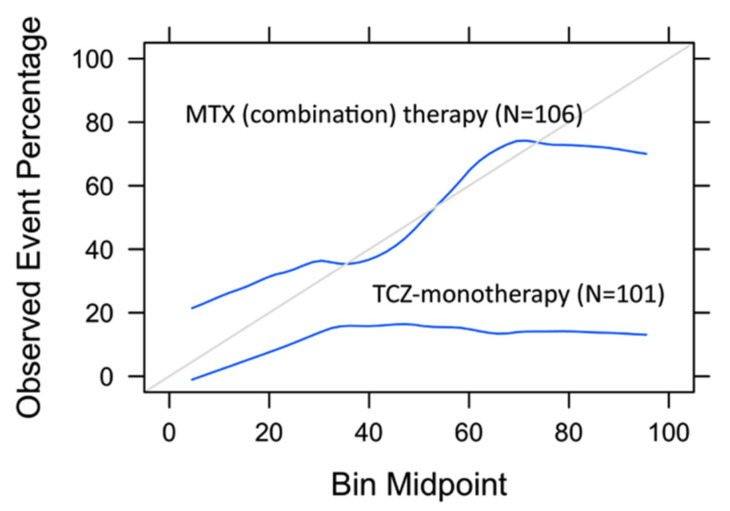
Calibration curves on test set of MTX (combination) therapy group and TCZ-monotherapy group. Logistic regression with six features (i.e., BMI, HAQ, smoking, ESR, TJC28, DMARD/corticosteroid use) was used to create calibration curves.

To assess whether the prediction model was specific for identification of insufficient responders to MTX (combination) therapy, the logistic regression model with six features was assessed on the TCZ-monotherapy arm of U-Act-Early. This group consisted of 101 patients of which 16 patients (16%) were insufficient responders at 3 months (DAS28 > 3.2). Confusion matrices are presented in Supplemental Figure S3. Upon the application of the model to the TCZ-monotherapy group, an AUC of 0.73 (95% CI: 0.60–0.86) was reached (Supplemental Figure S4) with a power of 86%, which was not significantly different from the AUC of 0.78 (95% CI: 0.69–0.87) with a power of 99% in the MTX combination therapy group (*p* = 0.54). However, the sensitivity dropped significantly from 83% in the MTX combination therapy group to 69% in the TCZ-monotherapy group (*p* = 0.03). Additionally, the model was better calibrated on the MTX (combination) therapy group than on the TCZ-monotherapy group, in which the percentage of actual insufficient responders was largely overestimated (Figure 3). This was also confirmed in a logistic regression model assessing risk score, treatment group, and their interaction in relation to insufficient response on the complete dataset (Table S2). The interaction term was just insignificant (*p* = 0.09).

## 4. Discussion

In this study, we showed that logistic regression performed equally well compared to machine-learning algorithms such as LASSO, random forest, and XGBoost in the prediction of insufficient response to MTX in RA patients on a current dataset. This result is in accordance with a recent systematic review where no benefit was discovered for the use of machine-learning algorithms in clinical prediction models compared to logistic regression [26]. Nevertheless, the approach of data analysis used for machine learning could still be very useful. First, the machine-learning approach allows internal validation by splitting the data into a training and a test set, thus reducing overfitting. Second, feature importance plots are an easy way to quickly inspect the importance of (combined) predictors on the outcome. In addition, a larger number of features can be evaluated regardless of the number of cases. Furthermore, machine-learning algorithms such as XGboost or random forest may be superior if the relationship between features and the outcome is more complex (nonlinear).

To enable comparisons between algorithm performances on the test set, we compared performance measures at the same cut-off on the ROC curve, for which any cut-off could have been chosen. In this study, we chose the cut-off where most insufficient responders were correctly classified (highest sensitivity) and at least 60% of sufficient responders were correctly classified (specificity). However, the best threshold for the trade-off between sensitivity and specificity depends on the clinical goal, as previously discussed [2,7].

According to the feature plots, we made a selection and showed that all models could be reduced from 16 to 6 essential features for the prediction of insufficient response. The features included were TJC28/DAS28, HAQ, ESR/CRP, BMI, smoking, and DMARD/corticosteroid use. Importantly, to select features according to feature importance plots, these plots should be carefully interpreted. Highly correlated features could make one feature seem irrelevant while that is not necessarily the case. An example is the low position of DAS28 in the logistic regression feature importance plot, which is due to its strong correlation with its component TJC28 (r = 0.73). In this case, TJC28 and baseline DAS28 were interchangeable, hence in clinical practice either one of the two correlated features could be used in the model. The same holds for ESR and CRP.

Our dataset contains a relatively high proportion (50%) of insufficient responders at 3 months, which can be explained by the design of the U-Act-Early trial. First of all, MTX dosage in U-Act-Early was slowly increased, reaching a dosage of 25 mg/week only after 3 months, while this dosage was reached in the tREACH within 3 weeks. This resulted in more insufficient responders from the U-Act-Early trial at 3 months. Moreover, all U-Act-Early patients received MTX-monotherapy, which in turn meant significantly more insufficient responders on MTX alone. This was accounted for in the model using the feature “DMARD/corticosteroids use”.

The majority of baseline features selected by LASSO were clinical features (e.g., DAS28, HAQ, BMI, smoking) and were in accordance with features from a previously validated prediction model on the same cohorts [7]. Furthermore, the same predictors were previously identified by others [5,6,27,28,29], although results on the direction of the effect of baseline DAS28 have been conflicting [5,6,30]. The exclusion of erythrocyte folate by LASSO was surprising, as this feature was required for the high AUC in our previous published model [7]. It seems that baseline ESR/CRP, which were not included in our previous model, could be used instead of erythrocyte folate to retain a high predictive power. ESR/CRP levels are easier to acquire compared to erythrocyte folate, hence the inclusion of this predictor instead strongly facilitates model implementation. The fact that some features are interchangeable leads to multiple combinations of predictors with similar predictive power. This has the advantage that clinicians can choose to use a model based on the feature availability in their own dataset. The model with six clinical features described in this study was therefore also uploaded in *Evidencio*: https://www.evidencio.com/models/show/2415).

Lastly, we showed that the final logistic regression model with six features performed better on the MTX (combination) therapy group than on the TCZ-monotherapy group, suggesting specific prediction of insufficient response to MTX (combination) therapy. Unfortunately, erythrocyte folate and ABCC3 genotypes, involved in the MTX metabolism [31], were not available in the TCZ-monotherapy group; hence, their contribution to specific prediction to MTX combination therapy could not be assessed. Baseline CRP/ESR and TJC28 are more generic predictors for response, shown to be associated with TCZ response in RA patients (CRP/ESR) and with etanercept response (TJC28) in juvenile idiopathic arthritis (JIA) patients [32,33]. Nevertheless, even with these generic predictors, the sensitivity dropped significantly from 83% in the MTX (combination) therapy group to 69% in the TCZ-monotherapy group. Additionally, the calibration curves showed that the predicted and observed risks fairly match in the MTX combination therapy group, while predicted risks largely overestimate the actual number of insufficient responders in the TCZ-monotherapy group (Figure 3).

The strengths of this study are that algorithms were tested head-to-head in the same group enabling direct comparisons of algorithm performances. Additionally, the final model was assessed on an independent therapy group starting with TCZ without previous DMARD use, suggesting that the model specifically identified insufficient responders to MTX (combination) therapy. The main limitation was the relatively small number of patients included. It is noteworthy that the number of cases in the TCZ-monotherapy group was limited (*N* = 16), however the ROC curve for this group still had a power of 86%. We may have also missed new features that could potentially improve the prediction regarding MTX (combination) therapy (e.g., global DNA methylation [34]) because we were limited to data availability in all included cohorts. However, the clinical features currently included in the model are often readily available and commonly assessed, which eases the implementation of the model into clinical practice.

In conclusion, logistic regression and machine-learning algorithms were on par in the prediction of insufficient response to MTX (combination) therapy. The model could be reduced to six features and was specific for the prediction of insufficient response in a MTX (combination) therapy group.

## Figures and Tables

**Table 1:** List of baseline features that were included in the study.

ABCB1 genotypes AA vs. AG/GG
ABCC3 genotypes TT vs. TC/CC
Age, years
Alcohol (Never consumed: 0, Ever consumed: 1)
Anti-citrullinated protein antibody (ACPA, negative/positive)
Body mass index (BMI), kg/m^2^
C-reactive protein (CRP), mg/L
Disease activity score 28 (DAS28)
DMARD and/or corticosteroid use (no use: 0, use: 1)
Erythrocyte folate (nmol/L packed erythrocytes)
Erythrocyte-sedimentation rate (ESR), mm/first hour
Gender (male/female)
Health assessment questionnaire (HAQ)
Rheumatoid Factor (RF, negative/positive)
Smoking, never/former vs. current
Tender joint count 28 (TJC28)

List of features included in the study based on availability and clinical rationale. ABCB1 = ATP binding cassette subfamily B member 1; ABCC3 = ATP binding cassette subfamily C member 3; DMARD = disease-modifying antirheumatic drug.

**Table 2:** Baseline comparisons between sufficient and insufficient responders.

	Insufficient Responders (3-Month DAS28 > 3.2)	Sufficient Responders (3-Month DAS28 ≤ 3.2)	*p*-Value
N (%)	177 (49.9%)	178 (50.1%)	
Age, mean ± SD	54 ± 13	53 ± 15	0.35
Gender, male	50 (28.2%)	63 (35.4%)	0.18
Rheumatoid factor, positivity	108 (67.1%)	113 (70.6%)	0.57
ACPA positivity	122 (69.3%)	136 (76.4%)	0.17
Behandeling			
MTX + SSZ + HCQ + i.m. cortico	28 (15.8%)	45 (25.3%)	0.04 *
MTX + SSZ + HCQ + cortico per os	31 (17.5%)	45 (25.3%)	0.10
MTX + cortico per os	41 (23.2%)	36 (20.2%)	0.63
MTX	77 (43.5%)	52 (29.2%)	0.01*

* *p*-value < 0.05 was considered significant. MTX = methotrexate. SSZ = Sulfasalazine. HCQ = hydroxychloroquine. i.m. = intramuscular. Cortico = corticosteroids. Missing values: erythrocyte folate *N* = 71, ABCB1 *N* = 16, ABCC3 *N* = 15, RF *N* = 34, ACPA *N* = 1, BMI *N* = 3, HAQ *N* = 15, smoking *N* = 14, alcohol use *N* = 14, CRP *N* = 1.

**Table 3:** Results of the model performances on test set (*N* = 106).

	AUC (95%CI)	Sensitivity	Specificity	Accuracy	PPV	NPV
Logistic regression	0.77 (0.68–0.86)	0.81	0.60	0.71	0.67	0.76
LASSO	0.76 (0.67–0.85)	0.79	0.60	0.70	0.67	0.74
Random forest	0.71 (0.61–0.81)	0.66	0.64	0.65	0.65	0.65
XGBoost	0.70 (0.61–0.81)	0.75	0.60	0.68	0.66	0.71

The threshold was chosen according to the highest sensitivity where specificity was at least 0.60. Baseline features included in the model: ABCB1 genotype, ABCC3 genotype, age, alcohol use, ACPA status, BMI, CRP, DAS28, DMARD/cortico use, erythrocyte folate, ESR, gender, HAQ, RF positivity, smoking, tender joint count 28 (TJC28). Abbreviations: LASSO = least absolute shrinkage and selection operater, XGBoost = extreme gradient boosting, PPV = positive predictive value, NPV = negative predictive value.

**Table 4:** The model performances on test set (*N* = 106) after feature selection.

	AUC (95%CI)	Sensitivity	Specificity	Accuracy	PPV	NPV
Logistic regression	0.78 (0.69–0.87)	0.83	0.60	0.72	0.68	0.78
LASSO	0.77 (0.68–0.86)	0.79	0.60	0.70	0.67	0.74
Random forest	0.76 (0.66–0.85)	0.79	0.62	0.71	0.68	0.75
XGBoost	0.77 (0.67–0.86)	0.79	0.62	0.71	0.68	0.75

Included features after feature selection were TJC28, HAQ, BMI, smoking, ESR, DMARD/corticosteroid use.

## Data Availability

Data are available upon reasonable request.

## Supplementary Materials

The following are available online at https://www.mdpi.com/2075-4426/11/1/44/s1, Table S1: Algorithm performances on the training set (*N* = 249), Figure S1: ROC curve of models tested on test set (*N* = 106) after feature selection, Figure S2: Confusion matrices on test set (*N* = 106) after feature selection, Figure S3: Confusion matrix of logistic regression on TCZ-monotherapy group, Figure S4: ROC curves of performance of final logistic regression model on test set MTX combination therapy and on TCZ-monotherapy group, Table S2: Logistic regression on complete dataset (MTX combination + TCZ monotherapy).
