# Supplementary material for: Complex Machine-Learning Algorithms and Multivariable Logistic Regression on Par in the Prediction of Insufficient Clinical Response to Methotrexate in Rheumatoid Arthritis
Source: J Pers Med. 2021 Jan 14;11(1):44. doi: 10.3390/jpm11010044 (PMC7828730; doi:10.3390/jpm11010044)
Supplement: Supplementary file 1 [file jpm-11-00044-s001.pdf]

Supplementary Files

Table S1. Algorithm performances on the train set (N = 249)

Algorithm	AUC
Logistic regression	0.72
LASSO regression	0.73
Random Forest	0.71
XGBoost	0.73

Algorithms performances are the result of 10-fold cross validation on the train set. 50% were insufficient responders. Insufficient response was determined at 3 months (DAS28>3.2)

Supplementary Figure S1. ROC curve of models tested on test set (N=106) after feature selection.

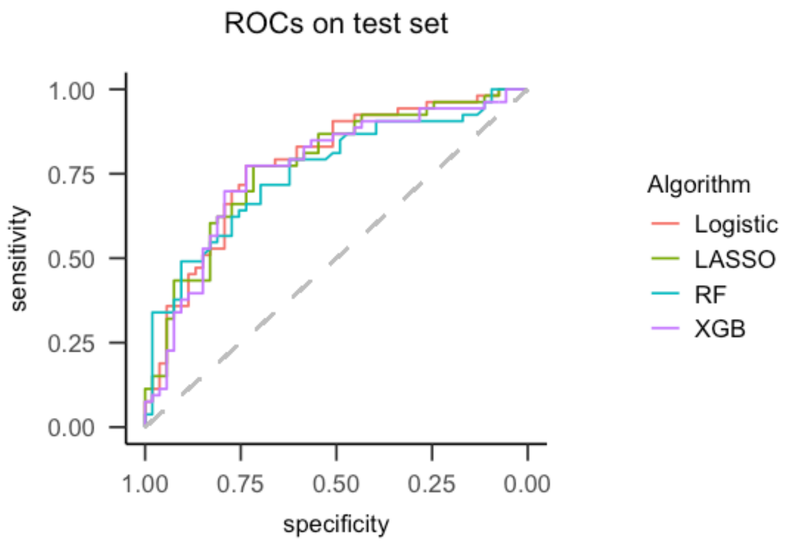

Features included in the model were: TJC28, HAQ, BMI, smoking, ESR, DMARD/cortico use
Logistic= logistic regression, LASSO= LASSO regression, RF= random forest, XGB= XGBoost

Supplementary Figure S2. Confusion matrices on test set (N=106) after feature selection. Features included in the model were: TJC28, HAQ, BMI, smoking, ESR, DMARD/cortico use. Thresholds were chosen where sensitivity was highest and specificity was at least 0.60. Grey-scale cells are correctly identified patients. IR=insufficient responder (DAS28 >3.2), R=sufficient responder (DAS28 ≤3.2)

Logistic regression

		Actual response	
		**IR (DAS28>3.2)**	**R (DAS28 ≤3.2)**
**Predicted**	**IR**	44	21
	**R**	9	32

LASSO

		Actual response	
		**IR (DAS28>3.2)**	**R (DAS28 ≤3.2)**
**Predicted**	**IR**	42	21
	**R**	11	32

Random Forest

		Actual response	
		**IR (DAS28>3.2)**	**R (DAS28 ≤3.2)**
**Predicted**	**IR**	42	20
	**R**	11	33

XGBoost

		Actual response	
		**IR (DAS28>3.2)**	**R (DAS28 ≤3.2)**
**Predicted**	**IR**	42	20
	**R**	11	33

Figure S3. Confusion matrix of logistic regression on TCZ monotherapy group

		Actual response	
		**IR (DAS28>3.2)**	**R (DAS28 ≤3.2)**
**Predicted**	**IR**	11	33
	**R**	5	52

IR=insufficient responder (DAS28 >3.2), R=sufficient responder (DAS28 ≤3.2)

Supplementary Figure S4. ROC curves of performance of final logistic regression model on test set MTX combination therapy and on TCZ monotherapy group.

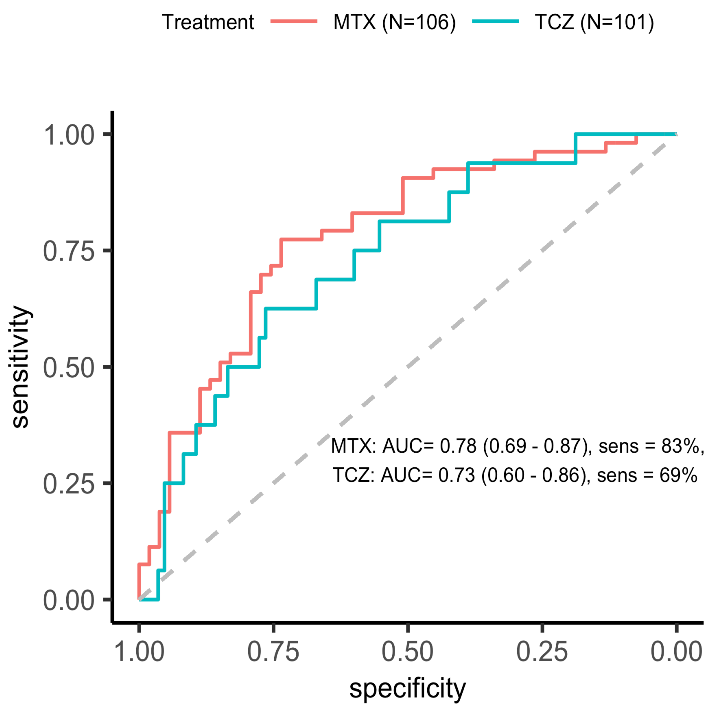

Features included in the model: TJC28, HAQ, BMI, smoking, ESR, DMARD/corticosteroid use. Sensitivity in both groups was determined at its highest point for which the specificity was at least 0.60.

Table S2. Logistic regression on complete dataset (MTX combination + TCZ monotherapy)

	Estimate	St. error	P-value
Intercept	-3.15	0.73	<1.73 x 10-5
Treatment (MTX combi)	0.86	0.82	0.29
Riskfactor	0.04	0.02	0.02
Treatment x riskfactor[#]	0.03	0.02	0.09

Interaction term between predictors. Predictors included in calculation risk factor: BMI, HAQ, smoking, ESR, TJC28, DMARD/cortico use.
